# Amyloid β and Amyloid Precursor Protein Synergistically Suppress Large-Conductance Calcium-Activated Potassium Channel in Cortical Neurons

**DOI:** 10.3389/fnagi.2021.660319

**Published:** 2021-06-03

**Authors:** Kenji Yamamoto, Ryo Yamamoto, Nobuo Kato

**Affiliations:** ^1^Department of Physiology, Kanazawa Medical University, Ishikawa, Japan; ^2^Department of Neurology and Clinical Research Center, National Hospital Organization Utano National Hospital, Kyoto, Japan

**Keywords:** amyloid precursor protein, amyloid β, Alzheimer’s disease, Homer1, BK channel

## Abstract

Intracellular amyloid β (Aβ) injection suppresses the large-conductance calcium-dependent potassium (BK) channel in cortical pyramidal cells from wild-type (WT) mice. In 3xTg Alzheimer’s disease (AD) model mice, intraneuronal Aβ is genetically programed to accumulate, which suppresses the BK channel. However, the mode of BK channel suppression remained unclarified. The present report revealed that only one (11A1) of the three anti-Aβ-oligomer antibodies that we examined, but not anti-monomer-Aβ-antibodies, was effective in recovering BK channel activity in 3xTg neurons. Antibodies against amyloid precursor protein (APP) were also found to be effective, suggesting that APP plays an essential part in this Aβ-oligomer-induced BK channel suppression in 3xTg neurons. In WT neurons, by contrast, APP suppressed BK channels by itself, which suggests that either APP or Aβ is sufficient to block BK channels, thus pointing to a different co-operativity of Aβ and APP in WT and 3xTg neurons. To clarify this difference, we relied on our previous finding that the scaffold protein Homer1a reverses the BK channel blockade in both WT and 3xTg neurons. In cortical neurons from 3xTg mice that bear Homer1a knockout (4xTg mice), neither anti-APP antibodies nor 11A1, but only the 6E10 antibody that binds both APP and Aβ, rescued the BK channel suppression. Given that Homer1a expression is activity dependent and 3xTg neurons are hyperexcitable, Homer1a is likely to be expressed sufficiently in 3xTg neurons, thereby alleviating the suppressive influence of APP and Aβ on BK channel. A unique way that APP modifies Aβ toxicity is thus proposed.

## Introduction

Alzheimer’s disease (AD) is a focus of global concern. Although no causal therapy is available yet, general anticipation is widespread toward potential interventions at the early stage, especially targeting the amyloidogenic pathway leading to amyloid β (Aβ) production (LaFerla et al., 2007; Perrin et al., 2009). Besides extracellular Aβ deposit, intracellular Aβ accumulation is documented in humans (Gouras et al., 2000; Takahashi et al., 2002), human iPS cell-derived neurons (Kondo et al., 2013; Ghatak et al., 2019) and rodent models (Oddo et al., 2003; Tomiyama et al., 2010; Ferretti et al., 2012; Iulita et al., 2014). In 3xTg model mice, intracellular accumulation proceeds Aβ deposit at the early stage of postnatal development, and therefore young pre-Aβ-deposit 3xTg mice provide a good model to represent an earlier stage of AD (LaFerla et al., 2007).

In pre-Aβ-deposit 3xTg mice, we have shown that a class of potassium channel, the large-conductance calcium-activated potassium (BK) channel, is suppressed in cortical pyramidal neurons. This channel, also called slo1, BK, maxiK, or K_*Ca*_1.1, is widely expressed and is regarded, if defective, to cause numerous diseases (Ghatta et al., 2006; Salkoff et al., 2006; Nardi and Olesen, 2008; Griguoli et al., 2016). The neuropsychological diseases relevant to BK channel include epilepsy, movement disorder, exacerbation and recovery of cerebral ischemic damage, intellectual disability, chronic neuropathic pain, alcohol use disorder, and AD (Contet et al., 2016). We have been analyzing the involvement of BK channel in AD pathogenesis by using a mouse model of AD (Yamamoto et al., 2011; Wang F. et al., 2015; Wang L. et al., 2015). Our hypothesis on how BK channel is involved in AD pathogenesis is that Aβ-induced BK channel suppression broadens action potentials and thereby enhances voltage-dependent calcium entry as we demonstrated, which would eventually collapse the calcium homeostasis and cause neuron death (Yamamoto et al., 2011). In WT mouse neurons, intracellular injection of Aβ indeed suppressed BK channel, widened spikes, and enhanced spike-induced calcium entry (Yamamoto et al., 2011).

It thus appears as if BK channel suppression in 3xTg mice were attributable to intracellular Aβ. However, this is not certain, since lines of evidence have suggested that amyloid precursor protein (APP) is also responsible for the toxicity attributed so far exclusively to Aβ in mouse models of AD, in most of which APP overproduction is prerequisite. First, increased cortical excitability and epileptic tendencies are reported in AD patients (Ferreri et al., 2003; Palop et al., 2007; Lam et al., 2017; Vossel et al., 2017) and model mice (Palop et al., 2007; Busche et al., 2012; Vossel et al., 2017; Zott et al., 2018, 2019). Some animal studies attributed hyperexcitability to elevated Aβ (Plant et al., 2006; Yamamoto et al., 2011; Scala et al., 2015), though APP overexpression itself is also regarded to be responsible (Born et al., 2014). Second, the binding between Aβ and APP is well documented (Lorenzo et al., 2000; Van Nostrand et al., 2002; Shaked et al., 2006; Fogel et al., 2014), and Aβ oligomer is shown to impair cognition only when bound to APP (Puzzo et al., 2017). Third, in knock-in model mice (FDD-KI and FBD-KI) for familial Danish dementia (FDD) and familial British dementia (FBD), neuronal defects occur only when sufficient levels of APP are supplied (Tamayev et al., 2011; Yao et al., 2019) and, more surprisingly, Aβ accumulation itself appears to be much less involved in these types of dementia than hitherto considered (Tamayev et al., 2012; Yao et al., 2019). These studies warn that the upstream of Aβ back to APP may be the focal point of AD pathogenesis. The present study revisited our previous proposal that the intracellular Aβ alone is responsible for BK channel suppression in 3xTg mouse neocortical neurons (Yamamoto et al., 2011), since a role played by APP might be critical as well.

## Materials and Methods

### Animals

All experiments were performed in accordance with the guiding principle of the Physiological Society of Japan and with the approval of the Animal Care Committee of Kanazawa Medical University. C57BL/6 wild-type mice (P26-39) were purchased from Sankyo Lab Ltd. (Toyama, Japan). Homomeric triple transgenic AD model mice (3xTg) with 129/C57BL6 hybrid background (Oddo et al., 2003), provided by Dr. LaFerla (University of California, Irvine), were kept in our in-house colony under an automatic day–night control (12:12 h), allowed to free access to food and water, and used at 4–5 months of age. Knockout mice that lack the Homer 1a isoform specifically but not the Homer 1b/c isoforms (H1a-KO) (Inoue et al., 2009), provided by Dr. Inokuchi (Toyama University, Toyama), were crossed with 3xTg mice (referred to as 4xTg), backcrossed more than five generations, and kept as homomeric in terms of both Homer1a and 3xTg. 4xTg mice were used at 4–5 months of age. Animals of both sexes were used for all the genotypes.

### Slice Preparations and Electrophysiology

**FIGURE 1:**
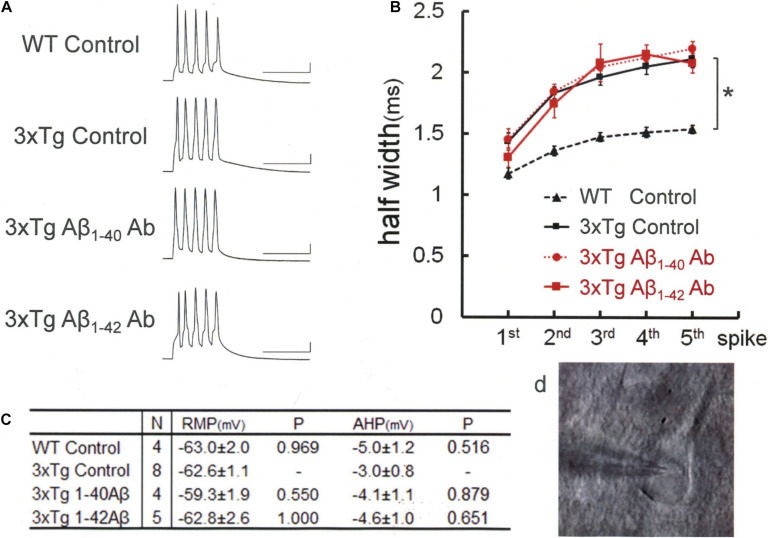
Intracellular injection of antibodies against Aβ monomer fails to rectify the spike broadening in 3xTg mice neocortex neurons. **(A)** Recordings of spike trains induced by brief current injections at 100 Hz into neurons in slices obtained from wild-type or 3xTg mice. Scale bars, 50 ms and 20 mV. **(B)** Averaged spike half-width during the five-spike train [black dashed line and triangle, WT Control; black solid line and square, 3xTg Control; red dotted line and circle, 3xTg Aβ_1__–__40_ antibody (G2-10); red solid line and square, 3xTg Aβ_1__–__42_ antibody (G2-11)]. With Aβ monomer antibodies, spike half-widths in 5th spikes were much the same as in 3xTg Control and significantly larger than in WT Control (*P < 0.001). Some of the error bars are too short to be seen clearly in this and the following figures. **(C)** Basic membrane properties of recorded neurons. Resting membrane potential (RMP) and afterhyperpolarization (AHP) were compared by using one-way ANOVA followed by Tukey HSD tests. No significant changes were induced by injecting antibodies or peptides. **(D)** A bright-field photomicrograph illustrating the arrangement of the pipette on a recorded pyramidal cell under microscope. The following Figures 2–5 are illustrated in the same convention.

Animals were decapitated under isoflurane anesthesia. The brain was dissected out and immersed in bathing medium (pH 7.4; 2–5°C) containing (in mM): NaCl, 124; KCl, 3.3; NaH_2_PO_4_, 1.3; NaHCO_3_, 26; CaCl_2_, 2.5; MgSO_4_, 2.0; and glucose, 20. Slices of the frontal cortex were prepared with a microslicer DTK1000 at 200 μm (Osaka, Kyoto, Japan). Slices were placed in a recording chamber on the stage of an upright microscope (BHWI; Olympus) with a 40× water-immersion objective (WPlanFl 40xUV). The chamber was continuously perfused with medium (25°C) bubbled with a mixture of 95% O_2_ and 5% CO_2_. Patch pipettes (resistance 4–10 MΩ) were filled with a solution (pH 7.3) containing (in mM) KCl, 7; K-gluconate, 144; KOH, 10; and HEPES, 10. Patch recording was done as previously described (Sun et al., 2011; Yamamoto et al., 2011). The photomicrograph in Figure 1D illustrates the arrangement of the pipette on a recorded pyramidal cell under a microscope. Whole-cell recordings were made from layer II/III pyramidal cells that had sufficiently negative resting membrane potential (RMP) (≤−55 mV) without spontaneous action potentials. RMP was recorded in the current-clamp mode (Axopatch 700 A, Axon Instruments, San Jose, CA, United States) and digitized at 10 kHz (Digidata 3827 and pCLAMP-8, Axon instruments). Membrane potentials in the current clamp mode were obtained by subtracting the bath potential from the whole-cell voltage reading. A train of five spikes at 100 Hz was evoked by a train of five depolarization current pulses (5 ms, 0.7 nA). In rare cases, the duration was decreased to at shortest 3 ms when 2 spikes were elicited during any one pulse, and was increased to at most 0.9 nA when there is a pulse failing to evoke a spike. After the fifth spike, spike afterhyperpolarization (AHP) was measured. We defined AHP as the most negative potential in the time window of 0–250 ms after the beginning of the first current pulse. A trough in this time window was adopted as AHP. Spike width measurement was done as previously described (Sun et al., 2011; Yamamoto et al., 2011).

### Peptides and Antibodies Used

Depending on the purpose of the experiments, peptides and various antibodies were purchased from the sources as follows, diluted at the concentrations specified below with the pipette solution, and injected intracellularly by diffusion: full-length APP (52 nM, OriGene), anti-Aβ_1__–__40_ monomer antibody (G2-10, 1 μg/ml; Millipore), anti-Aβ_1__–__42_ monomer antibody (G2-11, 1 μg/ml; Millipore), anti-Aβ_1__–__42_-oligomer antibodies (A11, 1 μg/ml; Millipore, 82E1, 1 μg/ml; IBL, Japan, 11A1, 1 μg/ml; IBL, Japan), anti-APP antibodies (22C11, 10 μg/ml; Millipore, A8717, 10 μg/ml; Sigma), and broad-band anti-amyloid antibody (6E10, 1 μg/ml; Covance). The peptide blocker of BK channel (charybdotoxin; CbTx) was bath-applied (50 nM; Alamone, Jerusalem, Israel). The recording session was started at least 5 min after whole-cell break in. Table 1 lists the antibodies used here.

**TABLE 1:** Antibodies used to block APP and Aβ.

Antibody	Antigen	Recognized epitope/region	Cross-reactivity	Clonality	Effect 3xTg	Effect 4xTg
G2-10	Aβ_1__–__40_ monomer	C terminus	Human and mouse	Mono	No	
G2-11	Aβ_1__–__42_ monomer	C terminus	Human and mouse	Mono	No	
11A1	Aβ oligomer	Oligomer toxic turn at 22/23	Human	Mono	Yes	No
A11	Aβ oligomer	Amyloid oligomer	Human and mouse	Poly	No	
82E1	Aβ generic	N terminus	Human	Mono	No	
6E10	APP and Aβ	16 aa’s* shared by APP/Aβ	Human and mouse	Mono	Yes	Yes
22C11	APP	N terminus	Human and mouse	Mono	Yes	No
A8717	APP	C terminus	Human and mouse	Poly	Yes	

aa*, amino acids.

### Data Analysis

Experimental data were obtained from 4 to 11 cells in neocortical slices of brains from mice of either sex. There were no differences in data between the sexes. Data are expressed as mean ± SEM. One-way ANOVA followed by *post hoc* Tukey HSD tests or Games–Howell tests, and *t* tests were used for statistics (SPSS v22, Japan IBM Ltd., Tokyo, Japan).

## Results

Our previous studies showed that intracellular Aβ renders the descending limb of action potentials less steep by blocking BK channels, but no AP4-sensitive K channels are blocked (Yamamoto et al., 2011). Relying on this finding, we adopted the spike broadening as an index of BK channel activity and, thereby, demonstrated that up- and down-regulation of BK channel activities are well correlated with cognitive amelioration and decline in 3xTg AD model mice, respectively, in our previous studies (Wang F. et al., 2015; Wang L. et al., 2015). These and other (Sun et al., 2011; Zhang et al., 2015) previous findings repeatedly confirmed the usefulness of spike width measurement as an assessment tool of the BK channel activity. In this report also, the width of spikes evoked by current injection in cortical pyramidal cells was adopted to evaluate the BK channel activity. We first confirmed that the width of spike was larger [Figure 1; Tukey HSD, *F*(3,17) = 14.772; *P* < 0.001] in 3xTg mouse neurons (5th spike; 2.11 ± 0.07 ms, *N* = 8 cells from 7 mice), which bear intracellular Aβ, than in WT neurons, which bear no Aβ (1.53 ± 0.05 ms, *N* = 4 cells from 3 mice; *P* < 0.001). By contrast, between 3xTg neurons and WT neurons, there were no significant difference in RMP [Tukey HSD, *F*(3,17) = 0.943, *P* = 0.442; 3xTg, −62.6 ± 1.1 mV, *N* = 8; Control, −63.0 ± 2.0 mV, *N* = 4; *P* = 0.969] or medium AHP [Tukey HSD, *F*(3,17) = 0.830, *P* = 0.496; 3xTg, −3.0 ± 0.8 mV, *N* = 8; Control, −5.0 ± 1.2 mV, *N* = 4; *P* = 0.516, Figure 1C], showing that the presence of intracellular Aβ affects the spike width without exerting a major impact on basic membrane properties that regulate spike generation.

Then, by relying on the spike width measurement, we characterized the efficacy of various anti-Aβ antibodies to counteract Aβ effects (Table 1). We postulated that some of the anti-Aβ-antibodies that we use would prevent Aβ from suppressing BK channels and would thereby narrow action potentials in 3xTg-AD mouse neocortical pyramidal neurons. We tested antibodies against Aβ monomers and three different kinds of anti-oligomer antibodies that recognize different types of epitopes within Aβ oligomers. Surprisingly, monomer antibodies against Aβ_1__–__40_ and Aβ_1__–__42_ failed to rectify the spike broadening in 3xTg mouse neocortex neurons (Figure 1). Intracellular injection of anti-Aβ_1__–__40_ antibody (G2-10) failed to render the width of the 5th spike narrower: 2.20 ± 0.06 ms, *N* = 4 cells from 2 mice; Control, 2.11 ± 0.07 ms, *N* = 8; Tukey HSD, *P* = 0.827. Anti-Aβ_1__–__42_ antibody (G2-11) also failed: 2.08 ± 0.08 ms, *N* = 5 cells from 4 mice; Control, 2.11 ± 0.07 ms, *N* = 8; Tukey HSD, *P* = 0.983. In 3xTg mouse neurons, these two antibodies did not alter RMP (G2-10, −59.3 ± 1.9 mV, *N* = 4; *P* = 0.550 vs. Control; G2-11, −62.8 ± 2.6 mV, *N* = 5; *P* = 1.000 vs. Control) and AHP (G2-10, −4.1 ± 1.1 mV, *N* = 4; *P* = 0.879 vs. Control; G2-11, −4.6 ± 1.0 mV, *N* = 5; *P* = 0.651 vs. Control, Figure 1C). Table 1 lists all the antibodies used in the present study.

**FIGURE 2:**
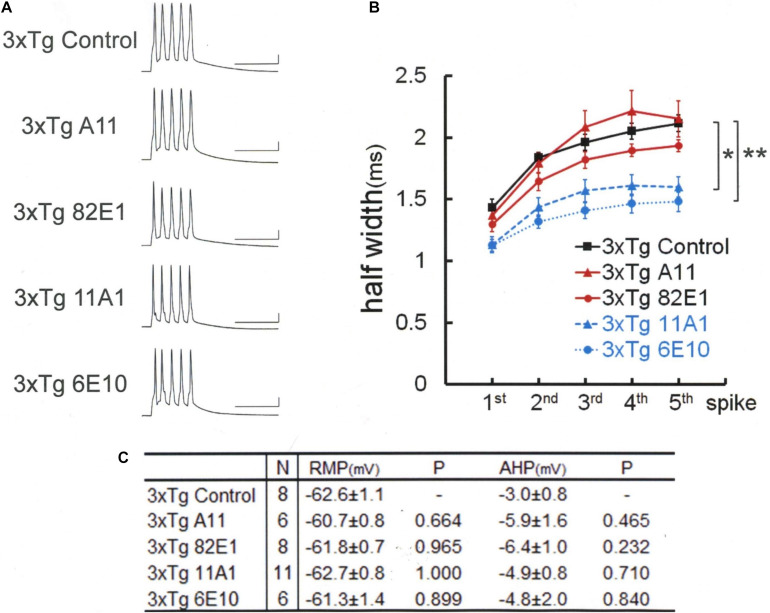
An Aβ-oligomer antibody (11A1) or 6E10 prevented spike broadening in 3xTg mouse neurons. **(A)** Specimen recordings of spike trains under the injection of anti-Aβ oligomer antibodies. Scale bars, 50 ms and 20 mV. **(B)** Averaged spike half-width during the five-spike train (black solid line and square, 3xTg Control; red solid line and triangle, 3xTg A11; red solid line and circle, 3xTg 82E1; blue dashed line and triangle, 3xTg 11A1; blue dotted line and circle, 3xTg 6E10). With 11A1 and 6E10, spike half-widths in 5th spikes were significantly smaller than in 3xTg Control (*P = 0.001, **P < 0.001). Note that 6E10 is reactive to a 16-amino-acid sequence common to Aβ, APP, and some APP C-terminal fragments. For the “3xTg Control” specimen recording and numerical data, those shown in Figure 1 are reproduced for clarity in this and following figures. **(C)** Basic membrane properties of recorded neurons. Convention is the same as in Figure 1.

Then, we used three different antibodies that recognize Aβ oligomer (Figure 2). One of these three antibodies (11A1) was effective in narrowing the spike width [Games–Howell, *F*(4,32) = 11.245 *P* < 0.001]: 11A1, 1.60 ± 0.08 ms, *N* = 11 cells from 2 mice; Control, 2.11 ± 0.07 ms, *N* = 8; *P* = 0.001. By contrast, a second one (A11; also called AB9234) was ineffective: A11, 2.15 ± 0.15 ms, *N* = 6 cells from 3 mice; Control, 2.11 ± 0.07 ms, *N* = 8; *P* = 0.998. Neither did the third one (82E1) exhibited any significant effect: 82E1, 1.93 ± 0.05 ms, *N* = 8 cells from 2 mice; Control, 2.11 ± 0.07 ms, *N* = 8; *P* = 0.254. It is reported that 82E1 recognizes the N terminus of Aβ monomer, oligomer and βCTF (β-C-terminal fragment), which is derived from APP by β cleavage, but not any part of APP (Horikoshi et al., 2004). Given this specific array of antigen recognition by 82E1 and our negative results with Aβ monomer antibodies, these findings obtained with the 3 antibodies reactive to Aβ oligomer suggest that βCTF fails to contribute to the spike broadening, whereas involvement of APP cannot be ruled out.

We next examined the effect of 6E10, which recognizes a 16-amino-acid sequence common to Aβ, APP, and βCTF (Figure 2). The blockade of spike broadening was completely achieved by injecting this antibody: 1.49 ± 0.08 ms, *N* = 6 cells from 4 mice; Control, 2.11 ± 0.07 ms, *N* = 8; *P* < 0.001. Here, again as with the data shown in Figure 1C, there is no difference across the groups in RMP [Tukey HSD, *F*(4,34) = 0.785, *P* = 0.543] and AHP [Tukey HSD, *F*(3,17) = 1.291, *P* = 0.293; Figure 2C]. Our findings so far indicate that not all but some forms of Aβ oligomer are responsible for BK channel suppression in 3xTg mouse neurons. Furthermore, it is suggested that APP, but not βCTF, may contribute to this BK channel suppression.

**FIGURE 3:**
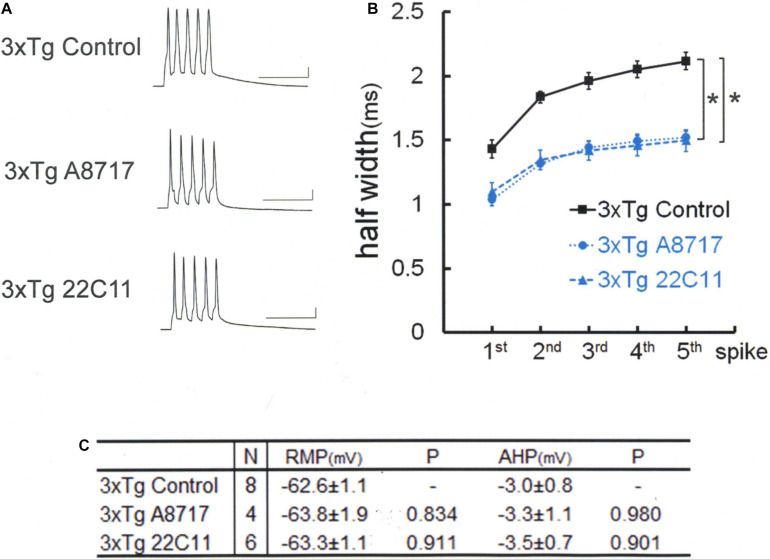
Amyloid precursor protein (APP) N-terminal (22C11) and C-terminal (A8717) antibodies reverse spike broadening in 3xTg mouse neurons. **(A)** Specimen recordings with anti-APP antibody injected. Scale bars, 50 ms and 20 mV. **(B)** Averaged spike half-width during the five-spike train (black line and square, 3xTg Control; blue dotted line and circle, 3xTg A8717; blue dashed line and triangle, 3xTg 22C11). Both 22C11 and A8717 are effective (spike half-widths in 5th spikes; *P < 0.001, vs. Control). **(C)** Basic membrane properties of recorded neurons. Convention is the same as in Figure 1.

Therefore, anti-APP antibodies were used to look whether APP blockade suffices to narrow the spike width in 3xTg neurons (Figure 3). An antibody recognizing the N terminus of APP (22C11) turned out to be effective in recovering the spike broadening [Tukey HSD, *F*(2,15) = 24.727, *P* < 0.001]: 22C11; 1.49 ± 0.08 ms, *N* = 6 cells from 2 mice; Control, 2.11 ± 0.07 ms, *N* = 8; *P* < 0.001. So did another antibody recognizing the C terminus (A8717): A8717; 1.52 ± 0.05 ms, *N* = 4 cells from 2 mice; Control, 2.11 ± 0.07 ms, *N* = 8; *P* < 0.001. There is no difference across the groups in RMP [Tukey HSD, *F*(2,15) = 0.189, *P* = 0.830] and AHP [Tukey HSD, *F*(2,15) = 0.095, *P* = 0.910; Figure 3C]. Since these antibodies do not recognize Aβ, it is suggested that blockade of either Aβ oligomer or APP (or partial peptide thereof) suffices to normalize the spike width in 3xTg neurons. These results indicate that both Aβ oligomer and APP are needed to suppress BK channels in 3xTg neurons, underscoring an important effect of APP.

**FIGURE 4:**
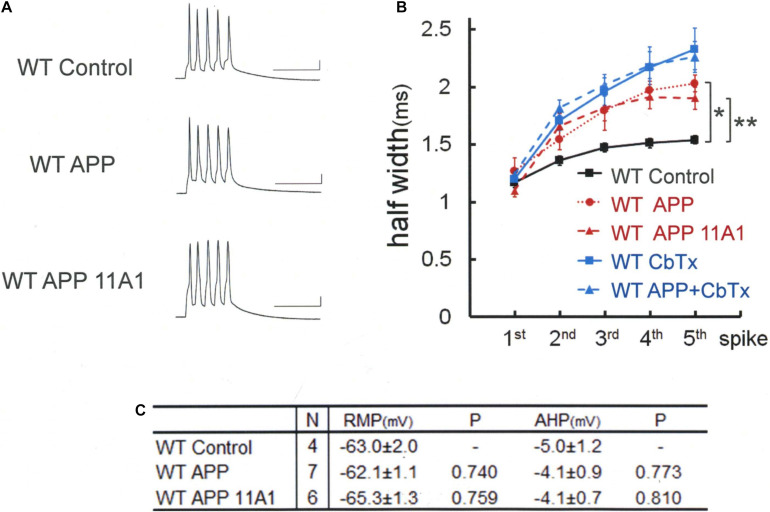
Intracellular application of APP broadens spike width independently of APP-derived Aβ oligomer. **(A)** Specimen recordings of spike trains in WT mouse neurons. Scale bars, 50 ms and 20 mV. **(B)** Averaged spike half-width during the five-spike train (black line and square, WT Control; red dotted line and circle, WT APP; red dashed line and triangle, WT APP 11A1). Both in the WT APP and WT APP 11A1 groups, spike half-widths in 5th spikes are significantly broadened compared with WT Control (*P < 0.001, **P = 0.004, respectively). For the “WT Control” specimen recording and numerical data, those shown in Figure 1 are reproduced for clarity. The blue lines indicate that the APP effect is occluded by a prior application of the charybdotoxin (CbTx), with the solid and dashed lines representing the CbTx alone and the APP + CbTx groups, respectively. **(C)** Basic membrane properties of recorded neurons. Convention is the same as in Figure 1.

We therefore tested whether the same APP effect is exerted in WT neurons as well (Figure 4). By injecting full-length APP into WT neurons, it was revealed that APP injection alone broadens the spike width [Tukey HSD, *F*(2,14) = 11.020 *P* = 0.001]: APP; 2.03 ± 0.05 ms, *N* = 7 cells from 2 mice; Control, 1.53 ± 0.05 ms, *N* = 4; *P* = 0.001. The effect of APP is not dependent on conversion of APP into Aβ, since the Aβ antibody 11A1, which we have shown is effective by itself in recovering BK channel activity, injected together with APP failed to interfere with the APP effect in WT neurons: APP + 11A1; 1.90 ± 0.10 ms, *N* = 6 cells from 2 mice; Control, 1.53 ± 0.05 ms, *N* = 4; *P* = 0.012. There is no difference across the groups in RMP [Tukey HSD, *F*(2,14) = 1.398, *P* = 0.28] and AHP [Tukey HSD, *F*(2,14) = 0.269, *P* = 0.768; Figure 4C]. Finally, we confirmed that it is by suppressing BK channel, as with Aβ, that APP broadened spikes (Figure 4B, blue lines). In the presence of the BK channel blocker charybdotoxin (CbTx, 50 nM) in the recording bath, the APP effect on spike width was occluded (5th spike; CbTx alone; 2.33 ± 0.18, *N* = 9 cells from 3 mice; APP + CbTx, 2.26 ± 0.14, *N* = 8 cells from 2 mice; *P* = 0.765; *t* test).

These findings so far indicate that either APP or Aβ suffices to suppress BK channels in WT neurons, whereas both are required in 3xTg neurons. Thus, BK channel recovery in 3xTg neurons is easier than BK channel suppression in WT neurons. In other words, the manner of BK channel blockade and recovery is not symmetrical between WT and 3xTg neurons. We hypothesized that this asymmetry may be accounted for by considering the involvement of the adaptor protein Homer1a. This hypothesis is based on our previous findings (Sakagami et al., 2005; Yamamoto et al., 2011) that (1) Homer1a injection activated BK channel in WT rat neocortical pyramidal cells; (2) Homer1a induced by electroconvulsive shock (ECS) reversed BK channel suppression in 3xTg neurons, which was abolished by injecting Homer1a antibody; and (3) BK channel suppression in Aβ-injected WT neurons was canceled out by Homer1a induced by ECS. Homer1a is an immediate early gene expressed by neural activity. AD model rodents including 3xTg mice have well been documented to show a higher neuronal excitability than WT animals (Palop et al., 2007; Busche et al., 2012; Wang F. et al., 2015; Wang L. et al., 2015; Zott et al., 2018, 2019). Indeed, our previous experiments by RT-PCR demonstrated that Homer1a mRNA expression in neocortex is higher in 3xTg than in WT mice (Wang F. et al., 2015). We therefore hypothesized that Homer1a expressed in 3xTg neurons may have partly recovered BK channels, causing the asymmetry of BK channel suppression.

**FIGURE 5:**
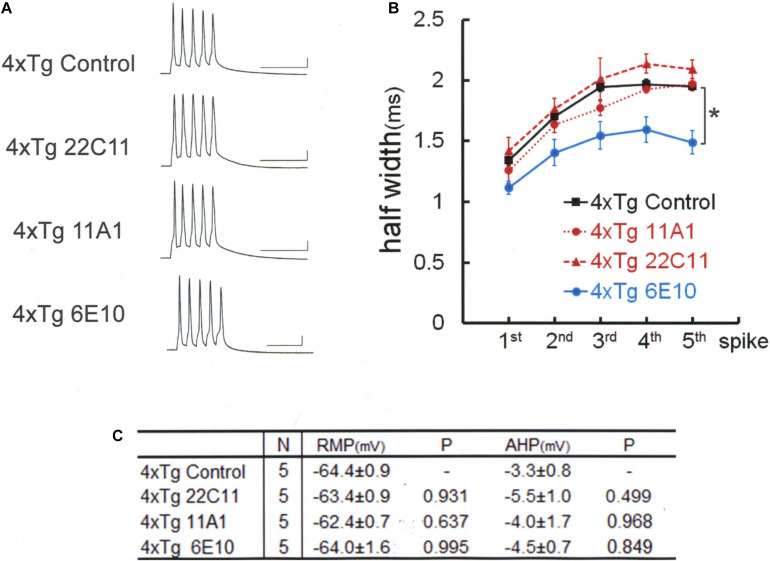
Spike broadening is blocked by 6E10, but not by anti-APP (22C11) or anti-Aβ oligomer (11A1) antibodies in 4xTg mouse neurons. **(A)** Specimen recordings of spike trains under the injection of anti-Aβ oligomer (11A1) or anti-APP (22C11) antibodies. Scale bars, 50 ms and 20 mV. **(B)** Averaged spike half-width during the five-spike train (black line and square, 4xTg Control; red dashed line and triangle, 4xTg 22C11; red dotted line and circle, 4xTg 11A1; blue line and circle, 4xTg 6E10). With 6E10, spike half-widths in 5th spikes were significantly smaller than in Control (*P = 0.025). With 22C11 or 11A1, there were no significant differences. **(C)** Basic membrane properties of recorded neurons. Convention is the same as in Figure 1.

To test this hypothesis, we used 4xTg mice, which are yielded by crossing 3xTg mice with Homer1a knockout mice (Figure 5). In 4xTg neurons, unlike in 3xTg neurons, the anti-Aβ antibody 11A1 failed on its own to recover BK channel [Games–Howell, *F*(3,16) = 9.134 *P* = 0.001]: 11A1; 1.97 ± 0.06 ms, *N* = 5 cells from 2 mice; Control, 1.95 ± 0.05 ms, *N* = 5 cells from 5 mice; *P* = 0.997. As with anti-Aβ 11A1 antibody, the anti-APP N-terminus antibody 22C11 was also ineffective. The spike width remained as large as without injection, failing to recover BK channel activity: 22C11, 2.09 ± 0.12 ms, *N* = 5 cells from 4 mice; Control, 1.95 ± 0.05 ms, *N* = 5; *P* = 0.717. This is in sharp contrast with the finding in 3xTg neurons that 22C11 completely normalized the spike width (Figures 3A,B). By contrast, a complete rectification of BK channel activity was achieved in 4xTg neurons by injecting the antibody 6E10, which blocks both Aβ and APP (Figure 5): 6E10; 1.49 ± 0.10 ms, *N* = 5; Control, 1.95 ± 0.05 ms, *N* = 5 cells from 4 mice; *P* = 0.025. As with 3xTg neurons, 4xTg neurons exhibited no differences across the groups in RMP [Tukey HSD, *F*(3,16) = 0.542, *P* = 0.660] and AHP [Tukey HSD, *F*(3,16) = 0.73, *P* = 0.549], despite injection of antibodies (Figure 5C). It was thus shown that the introduction of Homer1a knockout into 3xTg renders BK channels more vulnerable to Aβ and APP, since blocking either Aβ or APP is sufficient to recover those channels in 3xTg neurons but insufficient in 4xTg neurons, pointing to a stronger suppression of BK channels by each of Aβ and APP in 4xTg neurons.

## Discussion

**FIGURE 6:**
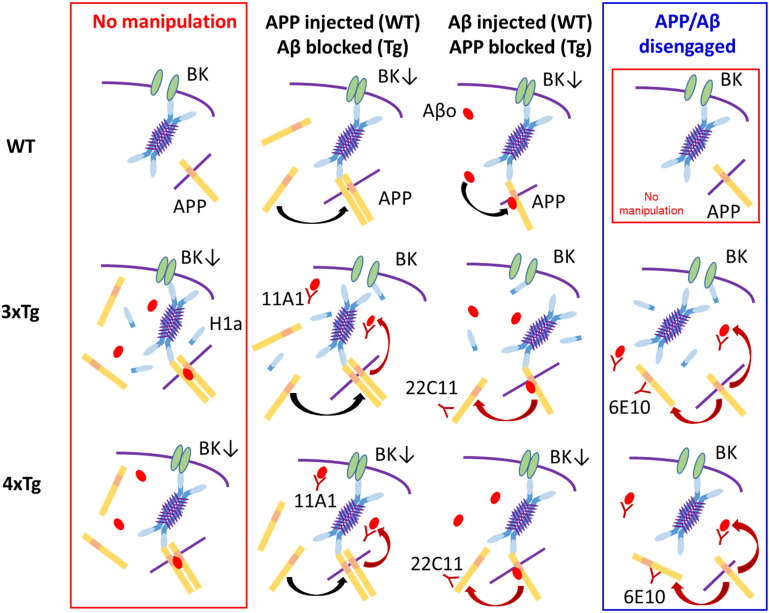
A tentative scheme illustrating the proposed mode of BK channel suppression and our interpretation based on the present experiments. The blue 4-armed structure represents the 4-mer of long Homers, which is considered to be the most stable form (Hayashi et al., 2009). Very short purple lines on the 4-mer signify the coiled-coil parts, which bind 4 long Homers to one another. The sky blue parts of the 4-mer represent the EVH domain, which binds to BK channel, APP and others. The isolated sky blue bar is the icon for Homer1a, which includes the EVH domain but lacks the coiled-coil domain and therefore competes with the 4-mer over BK channel. The yellow bars signify APP embedded in cytosolic or endoplasmic membrane (symbolized by the purple line) or floating in the free form. APPs tend to constitute dimers with or without the aid of Aβ oligomer (Aβo; the red oval). The downward arrow in “BK↓” symbolizes BK channel suppression (BK channel; green ellipses). (1) Top Row (WT neuron): *Leftmost*, In WT neurons, APP physiologically exists and embedded into endoplasmic or plasma membrane, but contacts the Homer 4-mer too weakly to persuade the 4-mer to suppress BK channel. *Second left*, With APP injected, excessive free APP dimerizes (black arched arrow) and strongly urges the 4-mer to block BK channels. *Second right*, With Aβ injected, Aβo binds to APP (black arched arrow). The Aβ-bound APP has a higher affinity and the 4-mer is more strongly persuaded to block BK channel. *Rightmost*, Identical to that in Leftmost. (2) Middle Row (3xTg neuron): *Leftmost*, In 3xTg neurons, the presence of abundant intracellular Aβ and APP urges formation of APP dimer-Aβ complex, strongly affecting the 4-mer and thereby blocking BK channels. Since 3xTg neurons are hyperexcitable, the activity-dependent expression of Homer1a continues but cannot reverse BK channel blockade in the presence of the APP dimer-Aβ complex. *Second left*, Because of APP overexpression, APP dimerizes (black arched arrow). With the anti-Aβ antibody 11A1 injected, the APP dimer-Aβ complex is disrupted (red arched arrow), weakening the potency of the 4-mer to block BK channels. The weakened 4-mer is disengaged by Homer1a, thereby relieving BK channel suppression. *Second right*, With the anti-APP antibody 22C11 injected, reduction of membrane-bound APP disrupts dimer formation (red arched arrow). However, Aβ is still excessive, and the Aβ–APP monomer binding is maintained. Overall, the suppression of the 4-mer on BK channel is weakened by dissolving the APP dimer-Aβ complex but kept effective by the Aβ–APP monomer binding. However, Homer1a is the competitor against the 4-mer over BK channel, and plays the decisive role, reversing BK channel blockade. *Rightmost*, With 6E10 that blocks both APP and Aβ, the 4-mer is weakened similarly (two red arrows) and disengaged from BK channel blockade by Homer1a. (3) Bottom Row (4xTg neuron): In 4xTg neurons, the states of the 4-mer, the APP dimer-Aβ complex, APP dimer formation, the Aβ–APP binding and BK channel blockade are the same as in 3xTg neuron. The effects of 22C11 and 11A1 are also the same, but the only difference resides in the absence of Homer1a. Therefore, the weakened 4-mer is not disengaged by Homer1a but continues to block BK channel (middle 2 panels). With 6E10 injected (rightmost), however, the weakening of the 4-mer would advance one-step further because of the double blockade of APP and Aβ, so that the 4-mer is disengaged even without Homer1a, thereby relieving BK channel. (4) Rightmost Column: APP monomer on its own cannot lead to BK channel suppression. (5) Leftmost Column: All neurons remain as they naturally are, with nothing injected. In 3xTg and 4xTg, affluence in both APP and Aβo forms the Aβo–APP dimer complex, which suppresses BK channel so strongly that Homer1a in the 3xTg neuron cannot rescue it. In 4xTg, the lack of Homer1a precludes the Homer1a-mediated rescue from the beginning. (6) Middle two columns: APP dimer or Aβo–APP monomer complex suppresses BK channels to a lesser extent than the Aβo–APP dimer complex. In 3xTg neurons, the relatively weaker complexes cannot overcome the counteraction of Homer1a. In WT and 4xTg neurons, where Homer1a is lacking or expressed little, even the relatively weaker complexes are sufficiently strong to suppress BK channels.

Dementia in AD is not necessarily ameliorated by preventing Aβ production. Rather, its origin APP or metabolic intermediaries may be more relevant than Aβ itself. Notwithstanding such recent views (Mitani et al., 2012; Tamayev et al., 2012; Egan et al., 2018; Esteve et al., 2019), we previously proposed that BK channels are suppressed by intracellular Aβ in 3xTg mouse neocortical neurons. The present study therefore set out to revisit this proposal by testing whether APP *per se* plays a more active role in BK channel suppression. Anti-APP or anti-Aβ-oligomer antibody alone was shown to reverse BK channel suppression in 3xTg neurons. With Homer1a knocked-out in 3xTg neurons (4xTg), antibody-mediated blockade of both APP and Aβ turned out to be required to rescue BK channel blockade, indicating that Homer1a antagonizes BK channel suppression by APP and Aβ. It is suggested that the Homer scaffolding critically affects the synergistic suppression of BK channels by Aβ and APP. Later in Discussion, we propose a hypothetical scaffolding of Aβ, APP, and Homers that may regulate BK channel activity (Figure 6).

We previously showed that intracellular injection of Aβ suppresses BK channel in WT neurons (Yamamoto et al., 2011). In the present experiments, this suppression was also achieved by injection of APP even under the co-existence of the anti-Aβ antibody 11A1, indicating that APP processing to Aβ plays only a minor role in BK channel suppression by APP in WT neurons. In 3xTg neurons, by contrast, antibody-aided blocking of either APP or Aβ is enough to rescue BK channel activity, suggesting that APP and Aβ work serially or concurrently in a synergistic manner. Given that the 11A1-induced disruption of APP processing to Aβ failed to prevent APP from blocking BK channel, the action of APP and Aβ appears to be concurrent rather than serial. In this connection, it is remarkable that Aβ attaches to APP at the sequence position from which Aβ would be yielded by β- and γ-cleavage, and this Aβ–APP complex even dimerizes (Shaked et al., 2006). It is further proposed that such a complex formation appears to be indispensable for Aβ toxicity (Shaked et al., 2006; Puzzo et al., 2017). Were such an Aβ–APP complex to mediate the BK channel blockade in 3xTg neurons, antibody blocking of either Aβ or APP would suffice to prevent this complex from suppressing BK channel. This is indeed what the present experiments have observed in 3xTg cortical pyramidal cells.

It is a well-known paradox that γ-secretase inhibitors, logically the most straightforward Aβ-lowering agents, are ineffective in ameliorating cognition in AD patients (Fleisher et al., 2008; Doody et al., 2013) and model mice (Mitani et al., 2012). A reconciling interpretation is that intermediary molecules upstream along the amyloidogenic pathway, including the precursor APP and βCTF (β-C-terminal fragment), may be more relevant to AD phenotypes than Aβ itself. Thus, the unsuccessful clinical trials of γ-secretase inhibitors, which sought Aβ lowering, appear to have turned attention toward the upstream of the amyloidogenic pathway. However, the clinical trial to lower βCTF by a β-secretase inhibitor also ended up with failure (Egan et al., 2018), directing more attention to APP, which is the substrate of β-secretase and therefore increased by β-secretase inhibitors. Consistently, the present report showed that antibody-led blockade of APP is sufficient to recover BK channel activity that is suppressed in 3xTg neurons. However, since APP is overexpressed in 3xTg neurons, the toxicity of APP on its own should not be overestimated. In neurons from most genetically modified model mice and human sporadic AD patients, Aβ is not negligible, so that there is a sufficient room for APP and Aβ to interact with each other. Although epileptogenic effects of APP have been documented in the mouse that overexpresses APP time-dependently (Born et al., 2014), Aβ deposit is not negligible in this model mouse either. For sure, the present report showed that APP injected into WT neurons has the same suppressing effect on BK channels as Aβ. In this case, however, injected APP is most likely localized in the cytosol, unlike APP overexpressed in model mouse neurons that is membrane bound, and may therefore be more prone to dimerization in the cytosol. By this way the toxicity of injected APP may be exaggerated, since APP dimers are reported to be more efficacious than monomers (Shaked et al., 2006). Overall, the present findings suggest that not just Aβ but also APP deserve attention. Other studies also suggest that APP, APP processing, and Aβ production are delicately balanced under mutual interference. First, when α-secretase mutations that co-segregate with late-onset AD are introduced into tg2576 AD model mice harboring APP Swedish mutation, anti-amyloidogenic α-cleavage of APP is shown to be reduced, with Aβ increased presumably by the enhancement of amyloidogenic β-cleavage (Suh et al., 2013). Moreover, an intrinsic α-secretase inhibitor is elevated in brains of patients with sporadic AD at various stages, and the knockout of this inhibitor in APP/PS1 AD model mice promotes anti-amyloidogenic α-cleavage of APP, thereby reducing Aβ (Esteve et al., 2019).

A distinct feature of the Aβ-induced BK channel suppression that we previously reported was its recovery by the adaptor protein Homer1a (Yamamoto et al., 2011). Homer1 constitutes a part of the scaffold protein family Homer and consists of the longer version Homer1b/c that can multimerize and form a scaffold attaching to various receptors like mGluRs and IP_3_Rs, and the shorter version Homer1a that lacks the coiled-coil domain, which would be required for multimerization, and therefore dominant-negatively prevents the scaffold formation by Homer1b/c (Brakeman et al., 1997; Kato et al., 1998). In line with the Homer1a-led recovery of Aβ-suppressed BK channel, this channel has a consensus motif at the C-terminus intracellular region that binds to Homer proteins (Tian et al., 2006). Since Homer1a and Homer1b/c are antagonistic, we hypothesized that a stronger scaffolding by the long Homer might exaggerate Aβ suppression on BK channel, which Homer1a relieves. All the more interesting is that APP is known to bind the long Homer proteins, which can sustain the scaffolding (Parisiadou et al., 2008; Kyratzi et al., 2015). It would then be plausible that Homer proteins and BK channels, together with APP and Aβ, may form a molecular complex that underlies BK channel suppression by APP and Aβ (Figure 6).

The present findings confirmed that the scaffolding protein Homer1a antagonizes Aβ-induced suppression of BK channels. In our preliminary experiments (Tajima, Liu, and Kato, unpublished observation), Aβ injection failed to suppress BK channels that were artificially expressed in HEK-293 cultured cells by the published method (Tajima et al., 2019; Liu et al., 2020). In agreement, Aβ application to recombinant BK channels embedded on their own in artificial membranes failed to decrease single channel conductance, with only a small reduction of open probability (Kawano et al., 2013). These findings are likely to rule out a direct binding of the injected Aβ to BK channel. Then their binding would have to be indirect in neurons. One likely way that Aβ inhibits BK channels is through the Homer protein scaffolding, since we have repeatedly confirmed that Homer1a, which antagonizes the Homer scaffolding, interferes with Aβ-led blocking of BK channel in neurons (Yamamoto et al., 2011; Wang F. et al., 2015; Zhang et al., 2015). Given that Homer1a antagonizes the scaffolding of the long Homers and rescues BK channel suppression, Aβ might indirectly bind BK channel via the long Homer scaffolding, thereby persuading the whole scaffolding into suppressing BK channel. In support, binding between long Homers and APP is documented, although the direct evidence came from Homer2 and Homer3, but not Homer1, as the representatives of the long Homers (Parisiadou et al., 2008; Kyratzi et al., 2015). As discussed, APP and Aβ bind each other and form a multimer complex, which would then enable Aβ and the long Homers to be associated via the Aβ–APP complex, thus forming an assembly consisting of BK channel, Aβ, APP, and long Homers. Homer1a would compete with long Homers over the binding site, namely, the proline-rich domain on the intracellular C-terminus domain of BK channel. We propose a tentative scheme describing our postulated scaffolding, though still very speculative (Figure 6).

The Homer-centered complex scaffolding that we propose here (Figure 6) is based on intracellular Aβ oligomer and its binding to APP. It was reported that scaffolding via extracellular Aβ oligomer is organized to aggregate mGluR5, perturbing calcium homeostasis (Renner et al., 2010). Their data documented intracellular assemblage of long Homers in parallel with mGluR5 clustering on the extracellular side, a reasonable phenomenon given that long Homer anchors mGluR5. Such Homer assembly attributable to extracellular Aβ oligomer might affect BK channels as well though they did not examine. Complementarily, the Homer scaffolding formed by intracellular Aβ and APP as we proposed here would exert the same effect on mGluR5 as did extracellular Aβ oligomer. By this way, extra- and intracellular Aβ oligomer may affect both mGluR5 and BK channel, and the present study has just focused on what occurred on the cytoplasmic side, not paying attention to the event on the extracellular side.

Finally, since injecting a pipette-filled APP into neurons does not occur naturally, how injected APP affects BK channels should be addressed. APP has a dual significance in neurons: one as the substrate of the amyloidogenic and non-amyloidogenic pathways, and the other as a signaling molecule like receptors or cell adhesion molecules (Deyts et al., 2016; Müller et al., 2017; Sosa et al., 2017). APP intracellular signaling, mediated by the intracellular domain, is a part of large-scale signaling system such as synaptic regulation, lysosome/endosome network functioning, protein internalization, and lipid transport (Deyts et al., 2016; Bukhari et al., 2017; Guénette et al., 2017; Müller et al., 2017; Nixon, 2017; Jiang et al., 2019; Montesinos et al., 2020). The intracellular domain takes more than one form of existence. First, it exists as the cytosolic terminal region of unprocessed APP or βCTF (also called C99), which is membrane-embedded and protruded into the cytosol from cell or vesicular membrane and, if vesicular-bound, moves along with the vesicles that accommodate the peptides. The second form of existence is a free form liberated after γ-cleavage (called AICD), which is also mobile. These two forms of the intracellular domain appear to be involved in parallel in different reactions because of the difference in total size and mobility, though there is a possibility that the free and membrane-inserted forms may be competitive over particular targets. Our intracellular injection of free APP here provided a third form of existence to the APP intracellular domain. Two ultimate destinies of free APP can be postulated. First, free APP may be in a dynamic equilibrium with membrane-bound APP and incorporated rapidly into cellular and vesicular membrane. Second, even if the injected free APP remains in cytosol, the C-terminus domain should at least work like liberated AICD or vesicle-bound C-terminus of APP/βCTF. The efficacy of C-terminus antibody that we showed can then be straightforwardly understood. Intracellular binding of N-terminus antibody might be artificial at glance, but this would nevertheless suppress the function or mobility of the whole APP including the C-terminus domain. The effects of N-terminus antibody that we observed here may be exerted by indirectly blocking the C-terminal domain. Overall, our interpretation is that intracellular injection of free APP is not completely artificial but is physiologically relevant albeit to a limited extent. Most importantly, the APP C-terminus domain is known to bind to the Homer EVH domain (Kyratzi et al., 2015), which we presume will persuade the long Homer tetramer to suppress BK channel (Figure 6). In this tetramer model, we propose that the injected APP may take over and carry out the physiological role played by membrane-bound APP in an augmented manner.

## Data Availability Statement

The original contributions presented in the study are included in the article/supplementary material, further inquiries can be directed to the corresponding author.

## Ethics Statement

The animal study was reviewed and approved by Animal Care Committee of Kanazawa Medical University.

## Author Contributions

KY and NK designed and performed the research, analyzed the data, and wrote the manuscript. RY performed the research.

## Conflict of Interest

The authors declare that the research was conducted in the absence of any commercial or financial relationships that could be construed as a potential conflict of interest.
